# Pneumomediastinum in a patient with severe Covid-19 pneumonia

**DOI:** 10.1590/0037-8682-0396-2021

**Published:** 2021-07-23

**Authors:** Chee Yik Chang

**Affiliations:** 1 Hospital Selayang, Medical Department, Selangor, Malaysia.

**FIGURE 1:**
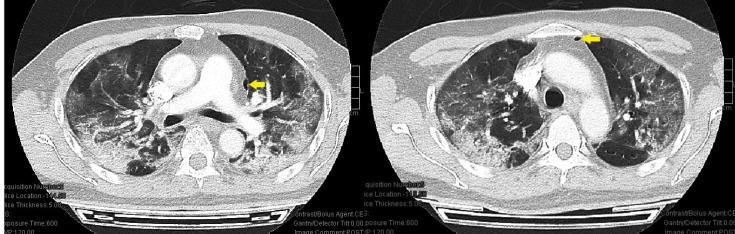
Computed tomography of the thorax showing patchy ground-glass densities and consolidative changes at bilateral dependent portions of the lungs and two foci of air locules at the anterior mediastinum, suggestive of pneumomediastinum (indicated by arrows).

A 57-year-old man with no prior medical illness complained of fever and cough for 6 days, followed by breathlessness 3 days later. He had close contact with his daughter-in-law, and was diagnosed with COVID-19 2 days prior. On arrival, he was febrile (body temperature = 38.5°C), not tachypneic, and had oxygen saturation of 99% on room air measured by pulse oximetry. Chest radiography showed ground-glass opacities in the bilateral lower zones. COVID-19 was confirmed by the detection of SARS-CoV-2 in nasopharyngeal and oropharyngeal swab samples using RT-PCR. In the ward, his clinical condition deteriorated with worsening of inflammatory markers and progressive hypoxemia that required oxygen supplementation via face mask on the third day of hospitalization and mechanical ventilation 3 days later. He responded well to intravenous dexamethasone and was extubated after 3 days. Computed tomography of the thorax revealed organizing pneumonia and pneumomediastinum (Figure 1). He still complained of cough but denied chest pain or worsening dyspnea. He received a tapering dose of oral prednisolone and underwent pulmonary rehabilitation in the ward. Pneumomediastinum was managed conservatively owing to general improvement. Oxygen support was weaned off, and he was discharged.

Pneumomediastinum is an uncommon complication of COVID-19 pneumonia. Its spontaneous form has been reported in COVID-19 patients without a history of mechanical ventilation^1^. Herein, the occurrence of pneumomediastinum was likely due to a combination of barotrauma and alveolar damage due to SARS-CoV-2 infection. Pneumomediastinum can develop after alveolar membrane damage and rupture, followed by air dissection through the bronchovascular sheath into the mediastinum^2^. It is regarded as a benign condition that requires only conservative management. Patients with pneumomediastinum should be monitored carefully for potential worsening of the disease^3^. 
